# Flow cytometry of bone marrow aspirates from neuroblastoma patients is a highly sensitive technique for quantification of low-level neuroblastoma

**DOI:** 10.12688/f1000research.53133.1

**Published:** 2021-09-21

**Authors:** Neha Jain, Shaista Sattar, Sarah Inglott, Susan Burchill, Jonathan Fisher, Andreea-Madalina Serban, Rebecca Thomas, Chris Connor, Niharendu Ghara, Tanzina Chowdhury, Catriona Duncan, Giuseppe Barone, John Anderson

**Affiliations:** 1 Great Ormond Street Hospital for Children, London, WC1N 3JH, UK; 2 Leeds Institute of Medical Research, St James University Hospital, Leeds, LS9 7TF, UK; 3 University College London Institute of Child Health, London, WC1N 3JH, UK

**Keywords:** neuroblastoma, paediatric cancer, bone marrow, flow cytometry, histology, RTqPCR, minimal residual disease

## Abstract

**Background:** Bone marrow involvement is an important aspect of determining staging of disease and treatment for childhood neuroblastoma. Current standard of care relies on microscopic examination of bone marrow trephine biopsies and aspirates respectively, to define involvement. Flow cytometric analysis of disaggregated tumour cells, when using a panel of neuroblastoma specific markers, allows for potentially less subjective determination of the presence of tumour cells.

**Methods:** A retrospective review of sequential bone marrow trephine biopsies and aspirates, performed at Great Ormond Street Hospital, London, between the years 2015 and 2018, was performed to assess whether the addition of flow cytometric analysis to these standard of care methods provided concordant or additional information.

**Results:** There was good concurrence between all three methods for negative results 216/302 (72%). Positive results had a concordance of 52/86 (61%), comparing samples positive by flow cytometry and positive by either or both cytology and histology.  Of the remaining samples, 20/86 (23%) were positive by either or both cytology and histology, but negative by flow cytometry. Whereas 14/86 (16%) of samples were positive only by flow cytometry.

**Conclusions: **Our review highlights the ongoing importance of expert cytological and histological assessment of bone marrow results. Flow cytometry is an objective, quantitative method to assess the level of bone marrow disease in aspirates.  In this study, flow cytometry identified low-level residual disease that was not detected by cytology or histology. The clinical significance of this low-level disease warrants further investigation.

## Introduction

Neuroblastoma is the most common extracranial solid tumour of childhood ( Xie, Onyskio and Morrison, 2018). A combination of stage of disease, patient age, tumour histology and tumour biology are used to risk stratify patients for treatment ( Monclair et al., 2008). Metastatic disease in patients more than 18 months of age places a patient in the high-risk category. Consequently, accurate staging at the time of diagnosis is critical. These patients receive multimodal treatment with chemotherapy, myeloablative chemotherapy and autologous stem cell rescue, surgery, radiation therapy and immunotherapy. Approximately 50% of those diagnosed with neuroblastoma have high-risk stage M disease, with poor overall survival of <50% ( Tas et al., 2020).

Consistent with recommendations from the International Neuroblastoma Risk Group (INRG), evaluation of both bone marrow cores (trephines) and aspirates is reported to most accurately detect bone marrow disease ( Aronica et al., 1998); a combination of bilateral cores and aspirates is associated with 94.7% sensitivity ( Parsons et al., 2017). Further, addition of immunohistochemistry to histological assessments can lead to increased inter-observer agreement ( Parsons et al., 2016). The INRG Staging System (INRGSS) defines bone marrow infiltration as any involvement of bone marrow aspirate or trephines detected by the either or combination of cytology, histology, and/or immunohistochemical techniques, with >10% bone marrow involvement being one of the criteria used to distinguish between stage M and Stage MS disease ( Monclair et al., 2008; Beiske et al., 2009; Burchill et al., 2017). A revision of the International Neuroblastoma Response Criteria (INRC) outlined that follow-up bone marrow samples with ≤5% involvement would represent minimal disease ( Park et al., 2017).

Alternative methods, including flow cytometry, immunocytology and quantitative reverse transcriptase polymerase chain reaction (RTqPCR), are currently being evaluated as more sensitive and specific methods for the detection of low-level disease than cytological or histological assessment ( Tsang et al., 2003; Corrias et al., 2004; Beiske et al., 2005; Ferreira-Facio et al., 2013; Uemura et al., 2019). Flow cytometry is a well-validated method of detecting bone marrow infiltration in haematological malignancies but its role in solid paediatric cancers is not established. Studies have demonstrated that flow cytometry can detect disease at lower levels than histopathology ( Komada et al., 1998; Tsang et al., 2003; Szantho et al., 2018). The first triple colour flow cytometry assay to detect neuroblastoma was developed in 1998 ( Komada et al., 1998). This has subsequently been optimised and today CD45-/CD56+/CD81+/GD2+ cells by flow cytometry have been accepted to represent neuroblastoma cells ( Swerts et al., 2004; Ferreira-Facio et al., 2013). Disialoganglioside (GD2) is detected in the vast majority of neuroblastoma cells, but also expressed by melanomas, gliomas and focally in rhabdomyosarcomas and osteosarcomas ( Beiske et al., 2005; Ferreira-Facio et al., 2013). Importantly, GD2 is not expressed by normal bone marrow cells ( Swerts et al., 2004). CD56 antibody is present on a subset of CD4+, and CD8+ T–cells and NK cells in peripheral blood, as well as neural derived cells and tumours ( Beiske et al., 2005). CD45 is present on all human leukocytes but absent on neuroblastoma cells. Using flow cytometry, Komada et al. (1998) were able to detect a single neuroblastoma cell in up to 1 × 10 ^4^/10 ^5^ mononuclear cells. Szantho et al. (2018) analysed 36 samples from 16 patients and concluded that flow cytometry was highly specific and more sensitive than immunohistochemistry, as more cells can be evaluated. However, other studies have suggested that flow cytometry is 10-fold less sensitive than immunocytology or quantitative reverse transcriptase polymerase chain reaction (RTqPCR) ( Swerts et al., 2004; Uemura et al., 2019).

The role of minimal residual disease (MRD) in neuroblastoma is increasingly under investigation, although its clinical utility is yet to be defined. In haematological malignancies PCR-based detection of MRD has become part of the routine method for risk stratification and ongoing monitoring of patients during treatment, with an escalation in treatment if there is inadequate MRD response. Corrias et al. (2004) used immunocytology to detect MRD in bone marrow of patients with localised neuroblastoma and found no significant difference in overall survival of patients with MRD compared to those without detectable MRD in bone marrow. In patients with metastatic disease there was no difference in overall survival by bone marrow disease detected by MRD using either immunocytology or PCR techniques. The Children’s Oncology Group (COG) also showed no difference in overall survival for patients with localised disease that had bone marrow involvement detected by immunocytology alone at diagnosis ( Seeger et al., 2000). In the same study, COG demonstrated a clear correlation between increasing tumour burden in bone marrow and poor event free survival in those patients with stage M disease, but no difference in survival if bone marrow infiltration was only detected by immunohistochemistry and not by cytology ( Seeger et al., 2000). Conversely, others have shown a poorer prognosis in those patients with neuroblastoma detected by flow cytometry but negative by immunophenotyping ( Popov et al., 2019) and poor overall survival in those with neuroblastoma detectable by RTqPCR after induction therapy ( Druy et al., 2018). These studies have been limited by the small number of analysed samples. Flow cytometry does have an advantage over immunocytology as it helps identify cases that have lost GD2 expression. This is increasingly important as future treatment concentrates on targeting GD2 expression either though GD2-antibodies or experimentally through GD2 targeting CART-cells ( Schumacher-Kuckelkorn et al., 2016).

In this study, our aim was to compare flow cytometry with the combination of histological and immunohistological assessment of trephines and cytological review of bone marrow aspirates, to determine if there is a difference in detection of positive results between the various methods and if flow cytometry can provide any additional information.

## Method

The study was performed as an internal evaluation of bone marrow results by flow cytometry in neuroblastoma, which had been introduced as a standard additional technique at Great Ormond Street Hospital, London in 2015. Samples from consecutive patients diagnosed with neuroblastoma at our institution between June 2015 to March 2018 were evaluated. Samples taken at any time point of treatment/surveillance were included in the review.

Disease stage for each patient was based on the INRG staging system (Monclair et al., 2009) and risk stratification was as the per the Children’s Cancer Leukaemia Group (CCLG) Guidelines ( Morgenstern et al., 2015). At each time point, samples for cytology of aspirate, flow cytometry of aspirate, and histology/immunocytology of trephine biopsy were taken from the left and/or right side, which were then grouped by side of collection. Bone marrow aspirates and trephines reports issued as part of routine of care were reviewed, which included morphological and flow cytometric assessment of aspirates, and morphology plus immunohistochemical staining of trephine biopsies. Flow cytometry was performed 12–60hrs post collection of bone marrow aspirates. Neuroblastoma cells were identified by using live/dead gating followed by identification of CD45 ^−^/Lin neg/CD56 ^+^/GD2 ^+^ stained populations.

**Figure 1.**
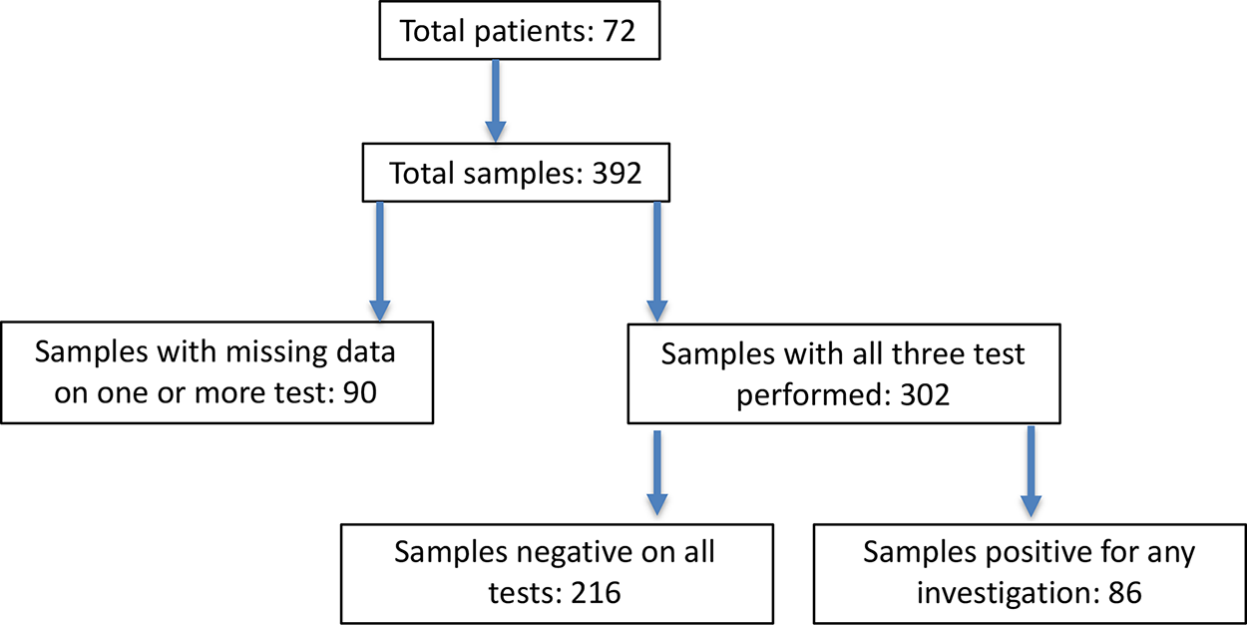
Numbers of cases and samples in the study.

For final analysis any patients with missing data for flow, aspirate or trephine analyses were excluded ( Figure 1). Any difference between the results of the trephine histology/immunohistochemistry, aspirate morphology, or flow cytometry were recorded. Significance testing was performed using unpaired t-test with Welch’s correction, with a p-value ≤ 0.05 considered as significant.

Eight patients also had bone marrow aspirates collected for RNA testing, performed by RTqPCR, as part of the European HR-NBL1/SIOPEN trial ( ClinicalTrials.gov registration number: NCT01704716) ( Viprey et al., 2014). The results from RTqPCR and flow cytometry analyses were compared, in order to establish if there are any correlations between the two assays. RNA was extracted and RTqPCR for the neuroblastoma mRNAs paired like homeobox 2B (PHOX2B) and tyrosine hydroxylase (TH) performed according to standard operating procedures ( Viprey et al., 2007, 2014). PHOX2B and TH are established neuroblastoma mRNAs ( Stutterheim et al., 2008; Brownhill and Burchill, 2017; Uemura et al., 2019).

For statistical analysis, the Log2 delta Ct values from the RTqPCR were converted to linear values for correlation with flow values by Pearson coefficient and correlation of flow with aspirate morophogy or trephine immunohistochemistry was performed using Welch's T test. Statistical analyis we performed using Prism software version 9.

## Results

A total of 392 bone marrow samples from 72 patients were analysed. Complete bone marrow, trephine and flow cytometry data was available for 302 samples ( Figure 1). RTqPCR results were available for 26 samples from 15 patients. A total of 15 samples from eight patients had both flow cytometry and RTqPCR data available (see *Underlying data*).

### Correlation between cytology, histology, and flow cytometry

**Figure 2.**
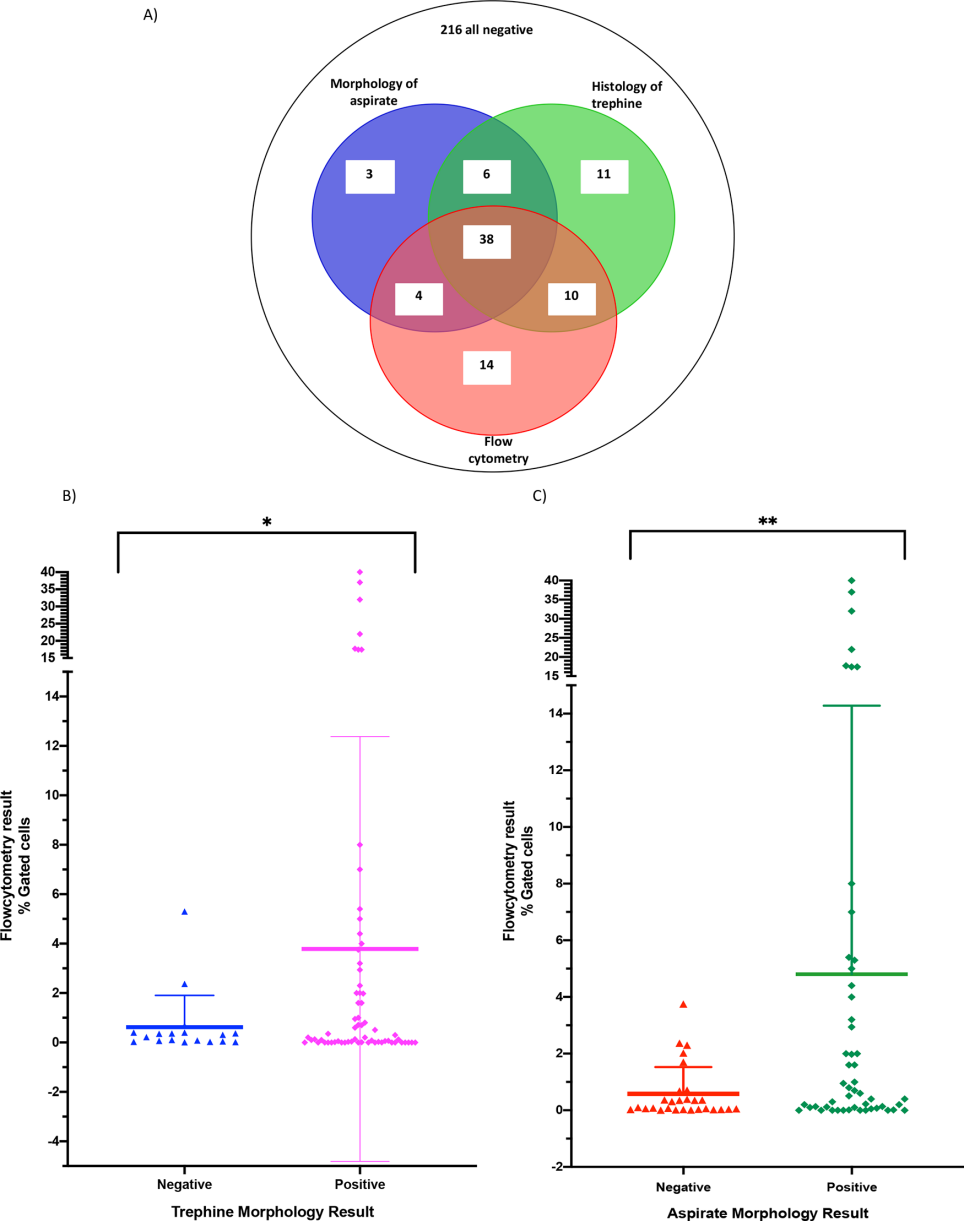
Comparison of positive results by flow cytometry, cytology and immunohistochemistry. A) Venn diagram of all positive cases. B) Negative trephine results in blue, positive trephine results in pink. 216 negative samples by all modalities excluded from analysis. Box and whisker plot showing the Mean and standard deviation of results *p-value 0.0056 by Welch’s t-test. C) Negative cytology results in red, positive cytology results in green. 216 negative samples by all modalities excluded from analysis. Box and whisker plot showing the Mean and standard deviation of results, **p-value 0.0027 by Welch’s t-test.

There was concordance in a negative result across all three modalities for 216/302 samples and a concordance of 38/86 for positive results across all three modalities ( Figure 2A), with a further 14/86 (16%) samples positive by flow cytometry and either cytology of aspirates or histology of trephine. Of the 86 samples that were positive by at least one test, 14/86 (16%) were positive by flow cytometry alone. Taken together, trephine and aspirate morphology detected 20/86 (23%) positives that were negative by flow cytometry (trephine only n = 11, cytology of aspirates only n = 3, both trephine and cytology of aspirates n = 6) ( Figure 2A).

Flow cytometry provides the additional benefit of allowing enumeration of the neuroblastoma cells within the bone marrow sample by calculating the positively gated events and negative gated events. We performed an absolute numerical comparison of flow cytometry results against the binary trephine and aspirate results ( Figure 2B and 2C) to determine if numerical flow cytometry results correlate with the aspirate morphology or trephine categorisation. Bone marrow samples that were positive by analysis of trephines were significantly more likely to be positive than negative on flow cytometry (p = 0.0027) and the same was true for samples positive for cytology (p = 0.0056), suggesting a good concordance between these modalities. When comparing trephine and flow cytometry, 18 samples were positive by flow cytometry but not positive on trephine histology. These samples had a percentage detection range of 0.0130% to 5.3% ( Figure 2B). Similarly, when comparing flow cytometry and cytology, there were 24 samples positive by flow cytometry, which were negative by cytology ( Figure 2C). These samples had a percentage detection range from 0.0041% to 3.75%. Therefore, flow cytometry of bone marrow aspirates detects low-level disease not reported after analysis of trephines or cytology of bone marrow aspirates.

### Patient disease course of flow cytometry-only positive samples

A total of 14 samples from nine patients were positive solely on flow cytometry. These patients represent potential cases where flow cytometry may be useful for detecting bone marrow disease below the combined threshold of cytology and trephines. All nine of these patients were diagnosed as high-risk ( Table 1). The level of disease detected by flow cytometry was low ranging from 0.008% to 2.37%. Only two patients (patient 5 and 7) had no radiological evidence of metastatic skeletal disease at the time of bone marrow sampling. Patient 5 had radiological localised disease and had bone marrow sample taken at diagnosis. This patient was treated as high-risk due having a *MYCN* amplified tumour and is now 42 months post diagnosis with no evidence of progression or relapse. Patient 7 had stage M high-risk neuroblastoma; the bone marrow sample was taken after completion of high dose chemotherapy with busulfan and melphalan. This patient is now 38 months post diagnosis with no evidence of relapse. Thus, the clinical follow up of these cases with low-level disease by flow cytometry does not provide any support for altering staging or treatment in such patients.

**Table 1.** Exclusively flow cytometry positive cases. L2 is localised unresectable disease and M denotes metastatic disease.

Participant	Laterality of sample	Flow cytometry %	Stage	Risk Stratification	MIBG/PET scan evaluation at time of bone marrow sample
1	R	0.02600	M	HR	Multiple skeletal metastasis
2	L	0.00820	M	HR	Multiple skeletal metastasis
2	R	0.07700			
3	L	0.04000	M	HR	Low grade uptake in skeletal metastasis
4	R	0.10000	M	HR	Multiple skeletal metastasis
4	L	0.06200			
5	R	0.31000	L2	HR	No skeletal metastasis
5	L	0.01300			
6	R	0.35000	M	HR	Multiple skeletal metastasis
6	L	0.68000			
7	L	0.36700	M	HR	No skeletal metastasis
8	L	2.37000	M	HR	Multiple skeletal metastasis
9	R	0.36100	M	HR	Multiple skeletal metastasis
9	L	0.02800			

### Correlation between flow cytometry and RTqPCR

**Figure 3.**
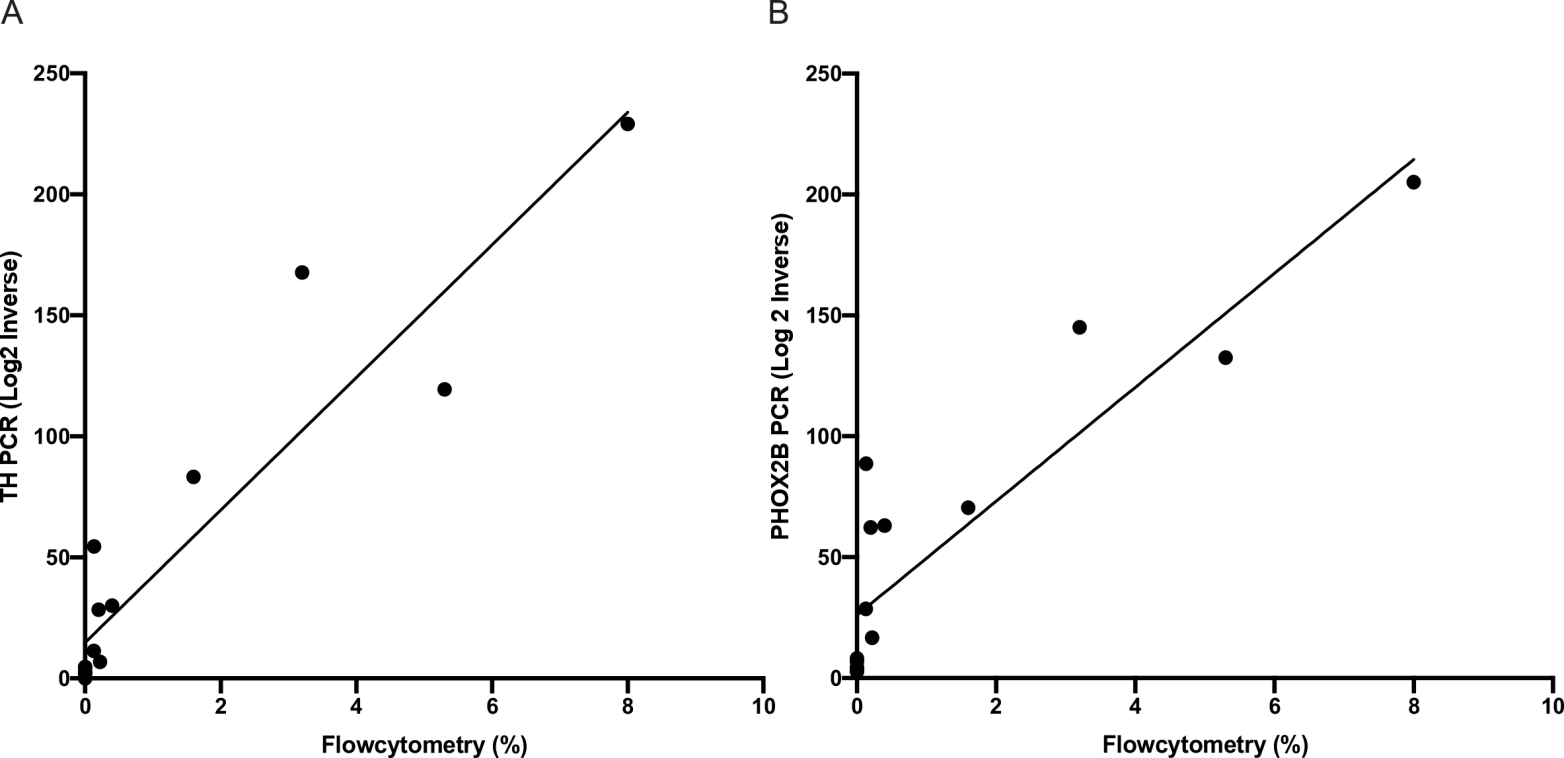
Correlation between reverse transcription quantitative polymerase chain reaction (RTqPCR) and flow cytometry results. Simple linear regression between RTqPCR and flow cytometry for fifteen samples A) R ^2^ co-efficient 0.8090 (p-value < 0.0001) and B) R ^2^ co-efficient was 0.8697 (p-value < 0.0001). Where PHOX2B is homeobox 2B, and TH is tyrosine hydroxylase.

To further evaluate the results of flow cytometry, we compared 15 samples from eight patients who had corresponding RTqPCR performed for mRNA using PHOX2B and TH markers. We performed simple linear regression modelling on RTqPCR and flow cytometry data for matched samples ( Figure 3). For PHOX2B the R ^2^ co-efficient was 0.8090 (p-value < 0.0001) and for TH R ^2^ co-efficient was 0.8697 (p-value < 0.0001). This excellent correlation between RTqPCR and flow cytometry further validates the flow cytometry results.

## Discussion and conclusion

In comparing flow cytometry, histology and cytology of aspirates results, our investigations show a good concordance across all three modalities for negative samples (72%). Taking positivity for either trephine and/or cytology of aspirates samples together, there is was also good concordance for positive results 52/86 (61%), though both flow cytometry (23%) or combination of histology/cytology (16%) did miss samples that were positive by the other modality. Furthermore, there was also good correlation between RTqPCR and flow cytometry results where both were available, providing further validation to flow cytometry results. Results positive by flow cytometry alone generally had low-level disease. There is increasing literature to suggest that clinically significant MRD in neuroblastoma can be detected using RTqPCR for neuroblastoma mRNAs ( Burchill et al., 2001; Viprey et al., 2014; Druy et al., 2018; van Wezel et al., 2016). These studies commonly include the two markers we have investigated, TH and PHOX2B mRNA. The persistence of bone marrow positivity is associated with poorer prognosis ( Horibe et al., 2001; Druy et al., 2018; Popov et al., 2019).

Flow cytometry is a routine test in diagnostic laboratories, which does require the development of expertise for analysis of results. Our results show some discordance between cytology/histology/flow cytometry. This discordance could be related to sampling differences, as different samples may be taken for analysis by various parts of diagnostic laboratories. Further, neuroblastoma cells have a propensity to aggregate. During flow cytometry analysis, clots are removed and samples filtered, which may lead to removal of some neuroblastoma aggregates. Bone marrow aspirates and trephine samples are not disaggregated, which may account for some disparity in results. Further, an element of subjectivity is present in the histological/cytology analysis of bone marrow trephines and aspirates, whereas flow cytometry provides an unequivocal characterisation of individual neuroblastoma cells.

Flow cytometry may be particularly useful for defining disease in patients who do not have adequate trephine biopsies or cells available for review on aspirates. It could serve as an additional quick and cost-effective tool for detection of low-threshold disease in patients with neuroblastoma. However, the presence of 20/86 samples with positivity by either cytology or histology analysis but no detectable neuroblastoma by flow cytometry, whilst may be accountable by sampling differences, highlights the importance of expert haematological and histopathological analysis of samples from these children. The clinical significance of low-level disease, detected using different methods, in neuroblastoma continues to be explored globally and remains to be seen.

## Data availability

### Underlying data

Open Science Framework: Flow cytometry analysis of neuroblastoma bone marrow. <registration DOI>

This project contains the following underlying data:

- De-identified HRNBL PCR and flow data.xlsx

<License statement>

https://osf.io/5c7ts/

## Ethic statement

The evaluation of results from bone marrow flow cytometry was a routine retrospective evaluation of standard of care procedures and not a formal research study. As such it did not require ethics committee approval. Consent for marrow aspirates and standard of care analysis was obtained from all patients using standard hospital consent procedures.

## Competing interests

No competing interests were disclosed.

## Grant information

The author(s) declared that no grants were involved in supporting this work.

## References

[ref1] Aronica PA Pirrotta V Yunis EJ : Detection of neuroblastoma in the bone marrow: biopsy versus aspiration. *J Pediatr Hematol Oncol.* 1998;20(4):330–334. 10.1097/00043426-199807000-00010 9703007

[ref2] Beiske K Ambros PF Burchill SA : Detecting minimal residual disease in neuroblastoma patients-the present state of the art. *Cancer Lett.* 2005;228:229–240. 10.1016/j.canlet.2005.02.053 15951104

[ref3] Beiske K Burchill SA Cheung IY : Consensus criteria for sensitive detection of minimal neuroblastoma cells in bone marrow, blood and stem cell preparations by immunocytology and QRT-PCR: recommendations by the International Neuroblastoma Risk Group Task Force. *Br J Cancer.* 2009;100:1627–1637. 10.1038/sj.bjc.6605029 19401690 PMC2696761

[ref4] Brownhill SC Burchill SA : PCR-based amplification of circulating RNAs as prognostic and predictive biomarkers – focus on neuroblastoma. *Pract Lab Med.* 2017;7:41–44. 10.1016/j.plabm.2016.04.003 28856217 PMC5575362

[ref5] Burchill SA Lewis IJ Abrams KR : Circulating neuroblastoma cells detected by reverse transcriptase polymerase chain reaction for tyrosine hydroxylase mRNA are an independent poor prognostic indicator. *J Clin Oncol.* 2001;19(6):1795–801. 10.1200/JCO.2001.19.6.1795 11251011

[ref6] Burchill SA Beiske K Shimada H : Recommendations for the standardization of bone marrow disease assessment and reporting in children with neuroblastoma on behalf of the International Neuroblastoma Response Criteria Bone Marrow Working Group. *Cancer.* 2017;123:1095–1105. 10.1002/cncr.30380 27984660

[ref7] Corrias MV Faulkner LB Pistorio A : Detection of neuroblastoma cells in bone marrow and peripheral blood by different techniques: accuracy and relationship with clinical features of patients. *Clin Cancer Res.* 2004;10:7978–7985. 10.1158/1078-0432.CCR-04-0815 15585633

[ref8] Druy AE Shorikov EV Tsaur GA : Prospective investigation of applicability and the prognostic significance of bone marrow involvement in patients with neuroblastoma detected by quantitative reverse transcription PCR. *Pediatr Blood Cancer.* 2018;65:e27354. 10.1002/pbc.27354 30007008

[ref9] Ferreira-Facio CS Milito C Botafogo V : Contribution of multiparameter flow cytometry immunophenotyping to the diagnostic screening and classification of pediatric cancer. *Plos One.* 2013;8(3):e55534. 10.1371/journal.pone.0055534 23472067 PMC3589426

[ref10] Horibe K Fukuda M Miyajima Y : Outcome prediction of molecular detection of minimal residual disease in bone marrow for advanced neuroblastoma. *Med Pediatr Oncol.* 2001;36:203–204. 10.1002/1096-911X(20010101)36:1<203::AID-MPO1049=3.0.CO;2-T 11464885

[ref11] Komada Y Zhang XL Zhou YW : Flow cytometric analysis of peripheral blood and bone marrow for tumour cells in patients with neuroblastoma. *Cancer.* 1998;82:591–599. 9452279

[ref12] Monclair T Broduer GM Ambros PF : The International Neuroblatoma Risk Group (INRG) Staging System: an INRG task force report. *J Clin Oncol.* 2008;27(2):298–303. 10.1200/JCO.2008.16.6876 19047290 PMC2650389

[ref13] Morgenstern DA Herd F Tweddle D : Guidelines for treatment of patients with low/intermediate risk neuroblastoma. *Children’s Cancer and Leukaemia Group (CCLG) Neurblastoma Special Interest Group (SIG).* 2015.

[ref14] Park JR Bagatell R Cohn SL : Revisions to the International Neuroblastoma Response Criteria: A consensus statement from the NCI-Clinical Trials Planning Meeting. *J Clin Oncol.* 2017;35(22):2580–2587. 10.1200/JCO.2016.72.0177 28471719 PMC5676955

[ref15] Parsons LN Gheorghe G Yan K : Improving detection of metastatic neuroblastoma in bone marrow core biopsies: a proposed immunohistochemical approach. *Pediatr Dev Pathol.* 2016;19:230–236. 10.2350/15-07-1676-OA.1 26491958

[ref16] Parsons LN Gheorghe G Yan K : An evidence-based recommendation for a standardized approach to detecting metastatic neuroblastoma in staging and bone marrow biopsies. *Pediatr Dev Pathol.* 2017;20(1):38–43. 10.1177/1093526616686253 28276294

[ref17] Popov A Druy A Shorikov E : Prognostic value of initial bone marrow disease detection by multiparameter ow cytometry in children with neuroblastoma. *J Cancer Res Clin Oncol.* 2019;145:535–542. 10.1007/s00432-018-02831-w 30603901 PMC11810268

[ref18] Schumacher-Kuckelkorn R Volland R Grandehandt A : Lack of immunocytological GD2 expression on neuroblastoma cells in bone marrow at diagnosis, during treatment, and at recurrence. *Pediatr Blood Cancer.* 2016;64:46–56. 10.1002/pbc.26184 27654028

[ref19] Seeger RC Reynolds CP Gallego R : Quantitative tumour cell content of bone marrow and blood as a predictor of outcome in stage IV neuroblastoma: a Children’s Cancer Group Study. *J Clin Oncol.* 2000;18(24):4067–4076. 10.1200/JCO.2000.18.24.4067 11118468

[ref20] Stutterheim J Gerritsen A Zappeij-Kannergieter L : PHOX2B is a novel and specific marker for minimal residual disease testing in neuroblastoma. *J Clin Oncol.* 2008;26(33):5443–5449. 10.1200/JCO.2007.13.6531 18838715

[ref21] Swerts K De Moerloose B Dhooge C : Detection of residual neuroblastoma cells in bone marrow: comparison of flowcytometry with immunohistochemistry. *Cytometry Part B (Clinical Cytometry).* 2004;61B:9–19. 10.1002/cyto.b.20019 15351977

[ref22] Szantho E Karai B Ivady G : Comparative analysis of multicolour flow cytometry and immunohistochemistry for the detection oof disseminated tumour cells. *Appl Immunohistochem Mol Morphol.* 2018;26:305–315. 10.1097/PAI.0000000000000519 28426528

[ref23] Tas ML Reedijk AMJ Karim-Kos HE : Neuroblastoma between 1990 and 2014 in the Netherlands: increased incidence and improved survival of high risk neuroblastoma. *Eur J Cancer.* 2020;124:47–55. 10.1016/j.ejca.2019.09.025 31726247

[ref24] Tsang KS Li CK Tsoi WC : Detection of micrometastasis of neuroblastoma to bone marrow and tumour dissemination to hematopoietic autografts using flow cytometry and reverse transcriptase-polymerase chain reaction. *Cancer.* 2003;97:2887–2897. 10.1002/cncr.11389 12767104

[ref25] Uemura S Ishida T Thwin KKM : Dynamics of minimal residual disease in neuroblastoma patients. *Front Oncol.* 2019;9:455. 10.3389/fonc.2019.00455 31214500 PMC6558004

[ref26] Van Wezel EM Decarolis B Stutterheim J : Neuroblastoma messenger RNA is frequently detected in bone marrow at diagnosis of localised neuroblastoma patients. *Eur J Cancer.* 2016;54:149–158. 10.1016/j.ejca.2015.11.007 26796600

[ref27] Viprey VF Corrias MV Kagedal B : Standardisation of operating procedures for the detection of minimal disease by QRT-PCR in children with neuroblastoma: quality assurance on behalf of SIOPEN-R-NET. *Europ J Cancer.* 2007;43(2):341–350. 10.1016/j.ejca.2006.08.007 17023157

[ref28] Viprey VF Gregory WM Corrias MV : Detection of mRNA in bone marrow and blood by RTqPCR predicts event-free and overall survival in children with stage 4 neuroblastoma at diagnosis; a SIOPEN Molecular Monitoring Group study. *J Clin Oncol.* 2014;32:1074–1083. 10.1200/JCO.2013.53.3604 24590653

[ref29] Xie L Onysko J Morrison H : Childhood cancer incidence in Canada: demographic and geographic variation of temporal trends (1992-2010). *Health Promot Chronic Dis Prev Can.* 2018;38(3):79–115. 10.24095/hpcdp.38.3.01 29537768 PMC6108034

