# Supplementary material for: Flow cytometry of bone marrow aspirates from neuroblastoma patients is a highly sensitive technique for quantification of low-level neuroblastoma
Source: F1000Res. 2021 Dec 6;10:947. Originally published 2021 Sep 21. [Version 2] doi: 10.12688/f1000research.53133.2 (PMC8825949; doi:10.12688/f1000research.53133.2)
Supplement: Peer review history

# Peer review: Reviewer response for version 2

**DOI:** 10.5256/f1000research.79864.r101983

**Published:** 2022-02-08

**Authors:** Toby Trahair

**Affiliations:** 1 Kids Cancer Centre, Sydney Children's Hospital, Randwick, NSW, Australia; 2 Children’s Cancer Institute, Lowy Cancer Research Centre, UNSW Sydney, Sydney, NSW, Australia; 3 School of Women’s and Children’s Health, UNSW Sydney, Sydeny, UNSW, Australia

I don't have further comments to make.

Is the work clearly and accurately presented and does it cite the current literature?

Yes

If applicable, is the statistical analysis and its interpretation appropriate?

Partly

Are all the source data underlying the results available to ensure full reproducibility?

Partly

Is the study design appropriate and is the work technically sound?

Partly

Are the conclusions drawn adequately supported by the results?

Partly

Are sufficient details of methods and analysis provided to allow replication by others?

Partly

Reviewer Expertise:

Neuroblastoma, acute lymphoblastic leukaemia, minimal residual disease detection

I confirm that I have read this submission and believe that I have an appropriate level of expertise to confirm that it is of an acceptable scientific standard.

# Peer review: Reviewer response for version 1

**DOI:** 10.5256/f1000research.56485.r94971

**Published:** 2021-10-26

**Authors:** Toby Trahair

**Affiliations:** 1 Kids Cancer Centre, Sydney Children's Hospital, Randwick, NSW, Australia; 2 Children’s Cancer Institute, Lowy Cancer Research Centre, UNSW Sydney, Sydney, NSW, Australia; 3 School of Women’s and Children’s Health, UNSW Sydney, Sydeny, UNSW, Australia

This is an interesting article that describes an analysis of bone marrow flow cytometry (FC) to identify bone marrow (BM) minimal residual disease (MRD) in consecutive patients diagnosed with neuroblastoma (NB) from a single centre, Great Ormond Street Hospital (GOSH). This is a laboratory-based comparative study focused on the comparison of NB BM MRD with morphology on BM aspirate, BM trephine and in a small number of cases to correlate with qRT-PCR for NB specific transcripts.

 MRD has an established role in the management of acute lymphoblastic leukaemia (ALL) and is routinely used in ALL clinical management. MRD in combination with clinical and genetic factors remains one of the most important clinical tools to guide therapy and risk stratification in both newly diagnosed and relapsed ALL ^1^^,^^2^^,^^3^^,^^4^^,^^5^^,^^6^^,^^7^. The two major techniques used to measure MRD in ALL are flow cytometry or quantitative PCR ^8^^,^^9^^,^^10^. The European Scientific foundation for Laboratory Hemato Oncology (ESLHO, https://eslho.org/about/) has established consortia to promote the innovation, standardisation, quality control and education of laboratory diagnostics including flow MRD (EuroFlow) ^10^ and quantitative PCR MRD (EuroMRD) ^8^^,^^9^. The International Neuroblastoma Risk Group (INRG) Task Force developed standard recommendations for the detection of residual neuroblastoma using immunocytology on BM cytospins or smears and/or qRT-PCR on BM ^11^. The INRG Task Force felt that the volume of bone marrow for analysis post-chemotherapy by flow cytometry may limit the sensitivity to detect residual NB to approximately 1 NB per 10 ^4^ BM cells ^11^, however, this is a level of sensitivity similar to what can be achieved using FC MRD in ALL MRD ^10^. However, the use of FC in the assessment of residual NB in BM remains clinically underdeveloped and it is a technology that may be easily and widely adapted to assessing treatment response in HR-NB.

 Currently, MRD and liquid biopsy are at an earlier stage of development in paediatric solid tumours and NB compared to ALL. The role of MRD in solid tumours continues to be explored within clinical research and/or clinical trials but is not currently used to modify risk classification or treatment. There are many evolving technologies that have the capacity to detect and quantify either residual tumour cells (e.g. immunocytology, flow cytometry, qRT-PCR for genes expressed in NB cells ^12^, DNA based assays to detect NB cells ^13^^,^^14^^,^^15^) or products secreted by tumour cells (e.g. cell-free tumour DNA ^16^^,^^17^, exosomal miRNA ^18^^,^^19^) which could be used to assess NB treatment response. So a challenge in the field is to clearly identify the methods and techniques of MRD/liquid biopsy which are prognostic and/or predictive in NB and which can be implemented in the clinical setting.

 The strengths of the current study are that it describes using FC to measure BM MRD in a consecutive cohort of 72 children with NB treated at Great Ormond Street Hospital. 392 BM samples were collected from these patients at various time points during their treatment. The study team has compared FC with immunocytology (BM aspirate) and histology (BM trephine). There were a limiting number of patients where qRT-PCR analysis was also available. In terms of the patient cohort, there is very limited detail on the clinical characteristics of the cohort, treatment or exact timing of the BM sample in relation to treatment received and clinical outcome. The only clinical information that is presented is for a subset of 9 patients with high-risk neuroblastoma (HR-NB) with FC positive marrows. In terms of FC methodology, the timing of FC varied from 12-60 hours post collection of the BM sample. The major experimental aim was a comparison of results between the different methodologies, so there is a focus on the comparative analysis of different techniques on individual samples. For negative samples (216) there was good concordance across all three techniques used (FC, trephine and aspirate morphology). The discordances arise in 86 samples which were found to be positive by at least one technique, with only 38/86 (44%) samples positive by all three assays. Each assay identified positive samples which were negative by both other techniques: FC 14/86 (16%), trephine histology 11/86 (13%), and 3/86 (3.5%) by aspirate. For the small number of patients who had both FC and qRT-PCR results, there was a good correlation between both techniques.

 Overall, the data shows that there is promise in using FC to assess NB MRD. As noted by the study team, FC is widely available and routinely used to assess haematologic malignancy. Undertaking these studies within the context of routine clinical practice is important as ultimately it can facilitate transfer into clinical practice. The EuroFlow, EuroMRD, and INRG consortia highlight the importance of the standardisation of sample collection, processing, workflow, analysis and reporting of samples ^8^^,^^9^^,^^10^. In this study, it’s not clear whether the time between collection and running the FC to assess NB MRD, between 12-60 hours, may have had an impact on the results. Specifically, are there differences in samples run at 12 versus 60 hours?

 In the GOSH cohort, it’s not clear how the FC assay performs with different input BM cell numbers and whether there were adequate cell numbers for each sample at each time point for the analysis. Assay performance and correlation are likely to be related to the amount of BM sample available for analysis and the levels of residual disease present within the sample. In ALL FC MRD, setting a threshold for the minimum number of BM cells to be analysed by FC is a key feature to permit sensitive detection of rare ALL cells after starting treatment ^10^. The performance of the FC, IC and qRT-PCR assays can be directly compared using spike-in experiments with NB cells and BM MNC, which, although not without limitations, allows an estimation of assay performance under different controlled conditions (e.g. BM cell number and dilution of NB cells).

 As discussed, it’s known that solid tumours and NB form clumps in the BM and it’s possible that a false negative FC result may occur from NB clumps being filtered out of the sample prior to the FC analysis (identified by the study team as a limitation) or as a consequence of NB being a “patchy” disease within the BM, so there may have been minimal or no NB at the site sampled by BMA, yet there may be clear evidence of BM disease at other sites on functional scanning (e.g. MIBG or FDG-PET CT scan). It would be helpful to understand how each of the marrow assessment methodologies compares with the measured INRC metastatic response ^20^ in the cohort.

 Whether there is additional value arising from FC NB MRD can’t really be assessed in this cohort due to the limited amount of clinical and laboratory data. It’s likely that the potential impact of NB MRD measured by any technique will be strongly influenced by the underlying genetics and risk classification of the individual patient. It’s known in ALL, MRD cutoffs are strongly influenced by the underlying ALL biology and genetics ^21^. It’s not unreasonable to hypothesise that the same holds true of NB, so the potential utility of NB FC BM MRD will be different in INRG Ms infants (low risk), in INRG M infants (intermediate risk) and in INRG M older children (high risk). An analysis of the data stratified by the patient’s risk classification may be informative, depending on the numbers. However, it’s possible to hypothesise that the role of FC to assess residual BM NB cells will be of most value in patients with high-risk disease.

 Although GOSH had introduced BM FC as a routine standardised assessment from 2015 onwards, the underlying objective(s) of assessing NB MRD by BM FC isn’t clearly stated in the submission. In managing ALL, assessing treatment response by MRD forms an integral component of the final ALL risk classification which occurs at the end of induction chemotherapy (i.e. after treatment has commenced). In ALL, rapid clearance of ALL and MRD negativity at the end of induction chemotherapy identifies patients with improved survival, and conversely slow ALL response with persisting high MRD levels at end induction identifies patients with decreased survival. In NB, risk classification is completed at diagnosis, prior to treatment initiation and is not modified by treatment response ^22^. Therefore, a potential role for NB MRD (measured by any methodology) to modify INRG risk stratification may not be likely. However, the clinical assessment of NB treatment response is critical and determines post-induction treatment allocation. So analogous to treatment response measurement in ALL, the tempo and completeness of treatment response in HR-NB is important and prognostic. HR-NB patients with inadequately responding disease have a poor chance of survival. One of the major clinical issues facing clinicians managing HR-NB is the early identification of patients with (i) inadequately responding disease or (ii) those who ultimately have a high risk of disease recurrence and poor survival. So standardised response assessment and MRD assessment at earlier time points in HR-NB induction chemotherapy might provide an opportunity to stratify patients to different treatments before the end of induction chemotherapy. Conversely, the role of MRD at later time points in treatment will be different, e.g. the early detection of relapsing disease in patients with a prior good response to treatment. So, in this context, whilst it’s important to assess the concordance of different laboratory assays which measure the same variable, it remains difficult to define the clinical significance of NB BM FC in this heterogenous cohort with samples taken at multiple time points and where the linked clinical data isn’t available. The value of this dataset will come into its own when it is more comprehensively linked and analysed with matching clinical and response information, specifically with clinically used and/or validated endpoints, such as INRC disease response, progression-free survival and overall survival.

 The data presented here represent an incremental step in using NB FC for MRD assessment. However, to take the field of liquid biopsy/MRD assessment forward in neuroblastoma, the ideal will be to prospectively define clear objectives for MRD assessment at different treatment time points (e.g. early or mid-induction versus later in therapy), collect appropriate samples at standardised time points, and directly compare multiple liquid biopsy/MRD methodologies against each other and also with the use of clinically validated endpoints.

Is the work clearly and accurately presented and does it cite the current literature?

Yes

If applicable, is the statistical analysis and its interpretation appropriate?

Partly

Are all the source data underlying the results available to ensure full reproducibility?

Partly

Is the study design appropriate and is the work technically sound?

Partly

Are the conclusions drawn adequately supported by the results?

Partly

Are sufficient details of methods and analysis provided to allow replication by others?

Partly

Reviewer Expertise:

Neuroblastoma, acute lymphoblastic leukaemia, minimal residual disease detection

I confirm that I have read this submission and believe that I have an appropriate level of expertise to confirm that it is of an acceptable scientific standard, however I have significant reservations, as outlined above.

## References

[1] : Prognostic value of minimal residual disease quantification before allogeneic stem-cell transplantation in relapsed childhood acute lymphoblastic leukemia: the ALL-REZ BFM Study Group. *J Clin Oncol* .2009;27(3) : 10.1200/JCO.2008.17.6065 377-84 10.1200/JCO.2008.17.6065 19064980

[2] : Molecular response to treatment redefines all prognostic factors in children and adolescents with B-cell precursor acute lymphoblastic leukemia: results in 3184 patients of the AIEOP-BFM ALL 2000 study. *Blood* .2010;115(16) : 10.1182/blood-2009-10-248146 3206-14 10.1182/blood-2009-10-248146 20154213

[3] : Effect of mitoxantrone on outcome of children with first relapse of acute lymphoblastic leukaemia (ALL R3): an open-label randomised trial. *Lancet* .2010;376(9757) : 10.1016/S0140-6736(10)62002-8 2009-17 10.1016/S0140-6736(10)62002-8 21131038PMC3010035

[4] : Late MRD response determines relapse risk overall and in subsets of childhood T-cell ALL: results of the AIEOP-BFM-ALL 2000 study. *Blood* .2011;118(8) : 10.1182/blood-2011-03-338707 2077-84 10.1182/blood-2011-03-338707 21719599

[5] : Augmented post-remission therapy for a minimal residual disease-defined high-risk subgroup of children and young people with clinical standard-risk and intermediate-risk acute lymphoblastic leukaemia (UKALL 2003): a randomised controlled trial. *Lancet Oncol* .2014;15(8) : 10.1016/S1470-2045(14)70243-8 809-18 10.1016/S1470-2045(14)70243-8 24924991

[6] : Treatment reduction for children and young adults with low-risk acute lymphoblastic leukaemia defined by minimal residual disease (UKALL 2003): a randomised controlled trial. *Lancet Oncol* .2013;14(3) : 10.1016/S1470-2045(12)70600-9 199-209 10.1016/S1470-2045(12)70600-9 23395119

[7] : Monitoring of minimal residual disease after allogeneic stem-cell transplantation in relapsed childhood acute lymphoblastic leukemia allows for the identification of impending relapse: results of the ALL-BFM-SCT 2003 trial. *J Clin Oncol* .2015;33(11) : 10.1200/JCO.2014.58.4631 1275-84 10.1200/JCO.2014.58.4631 25605857

[8] : Analysis of minimal residual disease by Ig/TCR gene rearrangements: guidelines for interpretation of real-time quantitative PCR data. *Leukemia* .2007;21(4) : 10.1038/sj.leu.2404586 604-11 10.1038/sj.leu.2404586 17287850

[9] : PCR Technology to Identify Minimal Residual Disease. *Methods Mol Biol* .2021;2185: 10.1007/978-1-0716-0810-4_6 77-94 10.1007/978-1-0716-0810-4_6 33165844

[10] : Standardized flow cytometry for highly sensitive MRD measurements in B-cell acute lymphoblastic leukemia. *Blood* .2017;129(3) : 10.1182/blood-2016-07-726307 347-357 10.1182/blood-2016-07-726307 27903527PMC5291958

[11] : Consensus criteria for sensitive detection of minimal neuroblastoma cells in bone marrow, blood and stem cell preparations by immunocytology and QRT-PCR: recommendations by the International Neuroblastoma Risk Group Task Force. *Br J Cancer* .2009;100(10) : 10.1038/sj.bjc.6605029 1627-37 10.1038/sj.bjc.6605029 19401690PMC2696761

[12] : Neuroblastoma mRNAs predict outcome in children with stage 4 neuroblastoma: a European HR-NBL1/SIOPEN study. *J Clin Oncol* .2014;32(10) : 10.1200/JCO.2013.53.3604 1074-83 10.1200/JCO.2013.53.3604 24590653

[13] : Whole-genome sequencing identifies patient-specific DNA minimal residual disease markers in neuroblastoma. *J Mol Diagn* .2015;17(1) : 10.1016/j.jmoldx.2014.09.005 43-52 10.1016/j.jmoldx.2014.09.005 25445214

[14] : Hypermethylated RASSF1A as Circulating Tumor DNA Marker for Disease Monitoring in Neuroblastoma. *JCO Precis Oncol* .2020;4: 10.1200/PO.19.00261 10.1200/PO.19.00261 32923888PMC7446415

[15] : Whole-genome sequencing facilitates patient-specific quantitative PCR-based minimal residual disease monitoring in acute lymphoblastic leukaemia, neuroblastoma and Ewing sarcoma. *Br J Cancer* .2021; 10.1038/s41416-021-01538-z 10.1038/s41416-021-01538-z 34471258PMC8810788

[16] : The pitfalls and promise of liquid biopsies for diagnosing and treating solid tumors in children: a review. *Eur J Pediatr* .2020;179(2) : 10.1007/s00431-019-03545-y 191-202 10.1007/s00431-019-03545-y 31897843PMC6971142

[17] : Shallow Whole Genome Sequencing on Circulating Cell-Free DNA Allows Reliable Noninvasive Copy-Number Profiling in Neuroblastoma Patients. *Clin Cancer Res* .2017;23(20) : 10.1158/1078-0432.CCR-17-0675 6305-6314 10.1158/1078-0432.CCR-17-0675 28710315

[18] : Serum-circulating miRNAs predict neuroblastoma progression in mouse model of high-risk metastatic disease. *Oncotarget* .2016;7(14) : 10.18632/oncotarget.7615 18605-19 10.18632/oncotarget.7615 26921195PMC4951313

[19] : Exosomal microRNAs from Longitudinal Liquid Biopsies for the Prediction of Response to Induction Chemotherapy in High-Risk Neuroblastoma Patients: A Proof of Concept SIOPEN Study. *Cancers (Basel)* .2019;11(10) : 10.3390/cancers11101476 10.3390/cancers11101476 31575060PMC6826693

[20] : Revisions to the International Neuroblastoma Response Criteria: A Consensus Statement From the National Cancer Institute Clinical Trials Planning Meeting. *J Clin Oncol* .2017;35(22) : 10.1200/JCO.2016.72.0177 2580-2587 10.1200/JCO.2016.72.0177 28471719PMC5676955

[21] : Genotype-Specific Minimal Residual Disease Interpretation Improves Stratification in Pediatric Acute Lymphoblastic Leukemia. *Journal of Clinical Oncology* .2018;36(1) : 10.1200/JCO.2017.74.0449 34-43 10.1200/JCO.2017.74.0449 PMC575632229131699

[22] : The International Neuroblastoma Risk Group (INRG) classification system: an INRG Task Force report. *J Clin Oncol* .2009;27(2) : 10.1200/JCO.2008.16.6785 289-97 10.1200/JCO.2008.16.6785 19047291PMC2650388

# Response

**Published:** 2021-11-26

**Authors:** JOHN ANDERSON

**Affiliations:** INSTITUTE OF CHILD HEALTH, UK

Thank you for an extremely comprehensive review of the article which constitutes by itself an important contribution to the discussion.

 No specific requests for clarification or further detail have been made apart from the request that the dataset (positivity in terms of flow, morphology, and trephines) be individually linked to clinical response to investigate the relative value of each technique as a response criterion. It is not clear if the reviewer is asking for such an analysis to be performed in the current dataset or suggesting a future prospective study.

 However, in our view it is not feasible to evaluate relative prognostic values of our markers in the current dataset for a number of reasons:

1. This was a retrospective not prospective study and there is marked heterogeneity of disease type and treatment.
2. A very limited number of discordant cases as now described in the revised manuscript (see the response to Reviewer 1).

It was never the intention of this study to explore flow cytometry as a biomarker. Our ambition was relatively limited; to determine whether the technique is feasible and has broad concordance with established techniques. In this, I think the study has been successful and it raises the possibility of flow cytometry being now evaluated prospectively.

# Peer review: Reviewer response for version 1

**DOI:** 10.5256/f1000research.56485.r94970

**Published:** 2021-10-22

**Authors:** Godelieve A. M. Tytgat

**Affiliations:** 1 Department of Pediatric Oncology, Princess Maxima Center for Pediatric Oncology, Utrecht, 3584 CS, The Netherlands

Flow cytometry of bone marrow aspirates from neuroblastoma patients is a highly sensitive technique for the quantification of low-level neuroblastoma.

 In this study, the authors undertake a very nice study to look at the sensitivity and specificity to study NBL cells in the bone marrow by flow cytometry. As there have been studies demonstrating the use of this technique, larger studies that actually compare this technique to other techniques on the same samples are lacking.

 In this work, the group studies the detection of NBL cells in a total of 392 samples of 72 patients. The set-up of the paper and the analyses are well done and well described. The article reads well and has a nice flow.

 There are some small questions/comments:

- In the methods, the authors describe that 8 patients had BM aspirates collected for RNA testing, and a few sentences later in the results they describe that PCR results were available for 26 samples from 15 patients. Please correct and align the actual numbers here.
- Furthermore, in the results, patient disease course of flow cytometry-only positive samples, the authors state that in their cohort the clinical follow-up of these cases with low-level disease by flow does not provide any support for altering staging or treatment in such patients. The study is not designed nor powered for such an analysis. We know that in patients with no BM metastases, detected by conventional techniques, low levels of mRNA or DNA and even NBL cells by flow can be detected, which has no impact on the outcome. So I agree with this conclusion and would like to emphasize that this can even be addressed stronger.
- The time points of sampling can be added to the table.
- Looking at Table 1, I wonder what were the results of earlier and later BM samples for patient 7? Did they become negative at a later time point for flow? Please add this information to the text.
- Finally, did the authors look at the paired samples of each patient (left and right side) and investigate the con- and discordances? Also, with respect to the different techniques?
- We test the BM involvement from 2 to 4 sides for each time point. How do the authors deal with the 2 sides? Did they merge the results? Please add this information to the paper.

Is the work clearly and accurately presented and does it cite the current literature?

Yes

If applicable, is the statistical analysis and its interpretation appropriate?

Yes

Are all the source data underlying the results available to ensure full reproducibility?

Yes

Is the study design appropriate and is the work technically sound?

Yes

Are the conclusions drawn adequately supported by the results?

Yes

Are sufficient details of methods and analysis provided to allow replication by others?

Yes

Reviewer Expertise:

Pediatric oncologist, translational research PIs

I confirm that I have read this submission and believe that I have an appropriate level of expertise to confirm that it is of an acceptable scientific standard.

# Response

**Published:** 2021-11-26

**Authors:** JOHN ANDERSON

**Affiliations:** INSTITUTE OF CHILD HEALTH, UK

Thank you for your detailed review.

- PCR numbers; sorry for the error in the methods part which has been corrected to state the following: “ *Patients with high risk neuroblastoma treated on  the European HR-NBL1/SIOPEN trial (ClinicalTrials.gov registration number: NCT01704716) (Viprey et al, 2014) also had bone marrow aspirates collected for RNA testing, performed by RTqPCR.”*  The results section is unchanged.
- Impact of low-level disease on disease outcome. We agree that this could be stated even more forcefully. The sentence has been changed to the following: *“Thus, the current study adds to existing understanding that in* *patients with no bone marrow metastases detected by conventional techniques, low levels of disease in the form of mRNA or DNA or neuroblastoma cells can be detected. There is no evidence that this low level disease can alter outcomes, and the clinical follow up of these cases in the current study does not provide any support for altering staging or treatment in such patients.”*
- Timing of sampling of positive flow results added to the table. This has been done.
- Patient 7 details: the following new test has been added: *“Patient 7 had stage M high-risk neuroblastoma with positive bone marrow morphology at diagnosis which became negative following induction. Following high dose chemotherapy with busulfan and melphalan high dose chemotherapy, the left sided aspirate was positive on flow cytometry but negative on morphology. Subsequent analyses were all negative.”*
- Concordance between left and right sites. We have added this information to the first results section with the following additional text: *“All comparisons involved evaluation of separate aspirate or trephine from left and right iliac crests respectively. We found low levels of intrapatient discordance between results from the two sites for both morphology (8 discordant results from 163 sampling episodes = 5%) and trephine (10/163 = 6%). Interestingly only one sampling event showed discordance in both aspirate and trephine.”*
